# 20 years of the Health and Environmental Surveillance Secretariat: 19 years of hospital epidemiological surveillance

**DOI:** 10.1590/S2237-96222024V33E20231407.EN

**Published:** 2024-03-11

**Authors:** Guilherme Almeida Elidio, Dirce Bellezi Guilhem

**Affiliations:** 1 Universidade de Brasília, Faculdade de Ciências da Saúde, Brasília, DF, Brazil

Dear editor,

Regarding Maciel’s editorial note,^1^ we would like to provide some commentary on one of the primary components of health surveillance in Brazil, aiming to enrich the debate on this topic.

The note extraordinarily recalls the strategy adopted 20 years ago: to consolidate, under the prism of a single ministerial secretariat, epidemiological surveillance programs for communicable and non-communicable diseases. In this context, it is worth highlighting hospital epidemiological surveillance. This surveillance was established nationally in 2004 and has since been operated by Hospital Epidemiology Centers (*Núcleos Hospitalares de Epidemiologia* - NHEs).^2^

The significance of hospital surveillance is immense; indeed, in the fight against COVID-19, the first suspected cases were reported by NHEs.^3^ These centers played a pivotal role in coping with the pandemic by implementing immediate measures to prevent intra-hospital transmission of the coronavirus, in addition to mitigating infection risks outside the hospital setting.

Recognizing the importance of this tool, the Ministry of Health established the National Hospital Epidemiological Surveillance Network (*Rede Nacional de Vigilância Epidemiológica Hospitalar* - RENAVEH), aimed at enabling knowledge, detection, preparedness and immediate response to public health emergencies occurring within hospital units.^4^ It is a tool of epidemiological surveillance with the mission of addressing all health events, as long as they remain within the hospital setting.

According to the records from the National Database of the National Health Establishment Registry (*Cadastro Nacional de Estabelecimentos de Saúde* - CNES) with active status as of November 2023, there are approximately 406,126 healthcare facilities in the country, of which 12.4% (50,176) are primary healthcare centers (PHC) or health centers with open access to the entire population.

Based on the successful experience of hospital epidemiological surveillance in responding to potential public health emergencies over the past 19 years, it is expected that the implementation of epidemiology centers in PHCs and health centers would strengthen health surveillance in fulfilling the detection, response and immediate monitoring of compulsory notifiable diseases, health conditions and events, similar to hospital epidemiological surveillance practices.

The institutionalization of epidemiology centers in these services can address the major challenges faced by the surveillance of each health condition, such as the data completeness in notification forms, timely investigation, timely vaccination blockade actions, isolation, timely notification, among others.^5^

Finally, it is worth highlighting the importance of maintaining, strengthening and expanding RENAVEH. We congratulate the author and Epidemiology and Health Services: Journal of the Brazilian National Health System (*Epidemiologia e Serviços de Saúde: revista do SUS* - RESS) for publishing this essential surveillance initiative for the Brazilian National Health System.
